# Photocatalytic Degradation of Single and Binary Mixture of Brilliant Green and Rhodamine B Dyes by Zinc Sulfide Quantum Dots

**DOI:** 10.3390/molecules26247686

**Published:** 2021-12-19

**Authors:** Peter A. Ajibade, Abimbola E. Oluwalana

**Affiliations:** School of Chemistry and Physics, University of KwaZulu-Natal, Private Bag, Scottsville, Pietermaritzburg 3209, South Africa; 217075609@stu.ukzn.ac.za

**Keywords:** ZnS, quantum dots, morphologies, photodegradation, brilliant green, rhodamine B, binary dye

## Abstract

We present the preparation of octadecylamine-capped ZnS quantum dots from bis(morpholinyldithiocarbamato)Zn(II) complex. The complex was thermolyzed at 130 °C in octadecylamine at different times, to study the effect of reaction time on the morphological and photocatalytic properties of the ZnS quantum dots. Powder X-ray diffraction patterns confirmed a hexagonal wurtzite crystalline phase of ZnS, while HRTEM images showed particle sizes of about 1–3 nm, and energy band gaps of 3.68 eV (ZnS–**1**), 3.87 eV (ZnS–**2**), and 4.16 eV (ZnS–**3**) were obtained from the Tauc plot for the ZnS nanoparticles. The as-prepared ZnS were used as photocatalysts for the degradation of brilliant green, rhodamine B, and binary dye consisting of a mixture of brilliant green-rhodamine B. The highest photocatalytic degradation efficiency of 94% was obtained from ZnS–**3** with low photoluminescence intensity. The effect of catalytic dosage and pH of the dyes solution on the photocatalytic process shows that pH 8 is optimal for the degradation of brilliant green, while pH 6.5 is the best for photocatalytic degradation of rhodamine B. The degradation of the binary dyes followed the same trends. The effect of catalytic dosage shows that 1 mg mL^−1^ of the ZnS nano-photocatalyst is the optimum dosage for the degradation of organic dyes. Reusability studies show that the ZnS quantum dots can be reused five times without a significant reduction in degradation efficiency.

## 1. Introduction

Rapid industrialization over decades has resulted in the release of organic contaminants into water bodies [1,2]. About 70,000 tons of industrial effluents produced annually are nondegradable organic compounds, and nearly 80% of the dyes among these organic contaminants are azo dyes [3,4]. The chemical structures of these dyes consist of aromatic rings that cannot be easily broken or biodegraded; they are also highly toxic, with mutagenic effects on aquatic life and humans [5,6]. An advanced oxidation process is one of the methods being used for wastewater treatment, especially the removal of organic contaminants [7,8,9]. An advanced oxidation process such as photocatalysis is based on the photogeneration of electron-hole pairs through a redox reaction at the semiconductor interface, to generate reactive species that interact with organic pollutants [10,11]. Interest in the use of semiconductor nanoparticles as photocatalysts for the degradation of organic dyes and subsequent removal from the environment is ascribed to the process being economical, without generating secondary pollutants [12,13,14]. TiO_2_ and ZnO are among the most commonly used semiconductors photocatalysts, but their applications are hindered by slow reaction kinetics due to the fast recombination of photogenerated electron-hole pairs [15]. Thus, there is a need to develop low-dimensional semiconductor materials with unique physiochemical, optical, and electronic properties [16,17,18,19,20]. Zinc-based nanomaterials are environmentally friendly at low concentration, and it is one of the most abundant trace metals in the human body [21]. Hence, zinc sulfide is among the semiconductor nanoparticles that can be used as a photocatalyst due to its high surface-area-to-volume ratio [22], which could enhance photon absorption at the nanointerface.

Xiao et al. [23] reported biofabrication of zinc sulfide nanoparticles, using the bacterium *Shewanella oneidenis* to partially degrade rhodamine B to N′,N′,N′,N′-tetraethylrhodamine after 3 h. Osuntokun et al. [24] tuned the morphology and optical properties of ZnS quantum dots prepared from the adduct of Zn(II) complexes. The ZnS quantum dots degraded ca. 75% methylene blue after 6 h. ZnS quantum dots prepared via co-precipitation-method photos catalytically degraded 80% of brilliant green dye after 80 min under solar-light irradiation [25]. Porous tube-like ZnS exhibited excellent degradation activity against Cr(VI) and reactive red X-3B under UV light with good reusability and stability after three cycles [26]. Biogenic ZnS quantum dots photodegraded 88% of organic dyes under sunlight after 8 h [27]. However, most of the reports on the use of ZnS quantum dots as photocatalysts showed that they suffer from low photodegradation efficiency [28,29,30]. Hence, the search for highly efficient ZnS quantum photocatalysts is a continuing effort. The size-dependent properties of nanomaterials depend most often on the synthetic approaches used for the preparation of the chalcogenide’s nanoparticles. Among synthetic techniques used for the preparation of metal sulfide nanoparticles, the use of single source precursors is an effective synthetic approach for the preparation of metal sulfide nanoparticles [31,32,33]. In this paper, bis(morpholinyldithiocarbamato)Zn(II) complex was used as a single-source precursor to prepare ZnS quantum dots. The precursor was thermolyzed in octadecylamine at 30 min, 1 h, and 2 h to evaluate the effect of thermolysis time on the optical, morphological, and photocatalytic properties of the as-prepared ZnS quantum dots. The photocatalytic potential of the as-prepared ZnS quantum dots was evaluated using brilliant green, rhodamine B, and binary dye mixture of brilliant green and rhodamine B. We also investigated the effect of the ZnS quantum dots’ catalytic dosage, scavengers, and pH on the photocatalytic degradation efficiency of the organic dyes by the as-prepared ZnS quantum dots.

## 2. Results and Discussions

### 2.1. Structural and Morphological Studies of ZnS Quantum Dots

Powder X-ray diffraction patterns (Figure S1-Supplementary Materials) of ZnS–**1** peaks at 22.87°, 24.41°, 27.76°, 33.15°, 42.87°, 47.40°, 52.46°, 56.05°, and 66.59° are indexed to the (100), (002), (101), (200), (102), (110), (103), (113), and (202) diffraction planes of the hexagonal wurtzite ZnS crystalline phase, while in the diffraction patterns of ZnS–**2** and ZnS–**3**, peaks at 23°, 27.73°, 23.36°, 43.05°, 46.74°, 51.87°, 55.70°, and 66.15° correspond to the (100), (101), (200), (102), (110), (103), (113), and (202) planes of the ZnS hexagonal wurtzite phase [JCPDS card no: 39-1363, a= 3.820 Å, c = 24.960 Å] [34,35]. The broad diffraction peaks at 23°, 28°, and 33° affirmed that the particle sizes of the as-prepared ZnS are zero-dimensional in the quantum dots’ nanometer range [11,36]. The XRD patterns show that diffraction peaks become sharper as thermolysis time increases [37].

**Figure 1:**
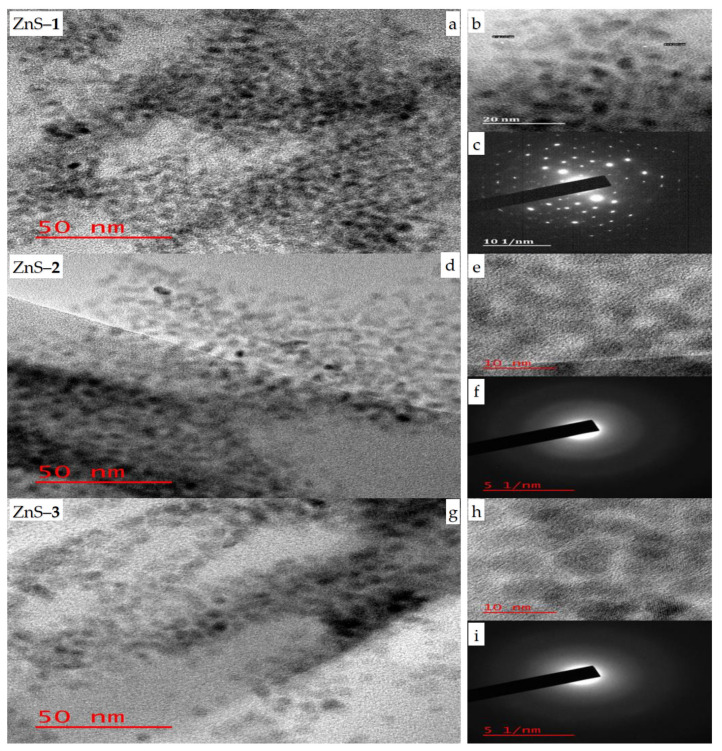
HRTEM images of ZnS–**1** (**a**), ZnS–**2** (**d**) and ZnS–**3** (**g**); lattice fringes, (**b**,**e**,**h**) and SAED patterns, (**c**,**f**,**i**) for zinc sulfide quantum dots.

HRTEM micrographs of the as-prepared ZnS quantum dots show that ZnS–**1** (Figure 1a) prepared at 30 min is spherical in shape with a particle size of 1.3 nm, while ZnS–**2** (Figure 1d), prepared at 1 h have particle sizes in the range of 1.08–3.42 nm, and the particle sizes of ZnS–**3** (Figure 1g), prepared at 2 h are in the range of 0.80–2.88 nm. The particle size distributions of the as-prepared ZnS quantum dots are presented in Figure S2. The interplanar spacing of the ZnS quantum dots’ (Figure 1b,e,h) lattice fringe of 0.22 nm corresponds to the (101) hexagonal plane [38]. The SAED patterns of the ZnS–**1** quantum dot (Figure 1c) confirmed that the as-prepared ZnS are highly crystalline in nature [34]. The SAED patterns of ZnS–**2** (Figure 1f) and ZnS–**3** (Figure 1i) consist of three blurred circular ring patterns, which indicate the presence of an amorphous layer that could be ascribed to the deposition of some capping agent on the surface during microscopic analyses. The SEM images (Figure S3) show cubic-like surface morphology for ZnS–**1** and ZnS–**3,** while ZnS–**2** has a rough surface morphology. The presence of zinc and sulfur in a semi-quantitative 1:1 ratio in the EDX spectra (Figure S4) confirms the existence of zinc sulfide quantum dots. The spectra also show carbon and oxygen ascribed to the octadecylamine capping agents. The presence of gold in the spectra is due to the material used for coating the samples to run the SEM-EDX analysis.

**Figure 2:**
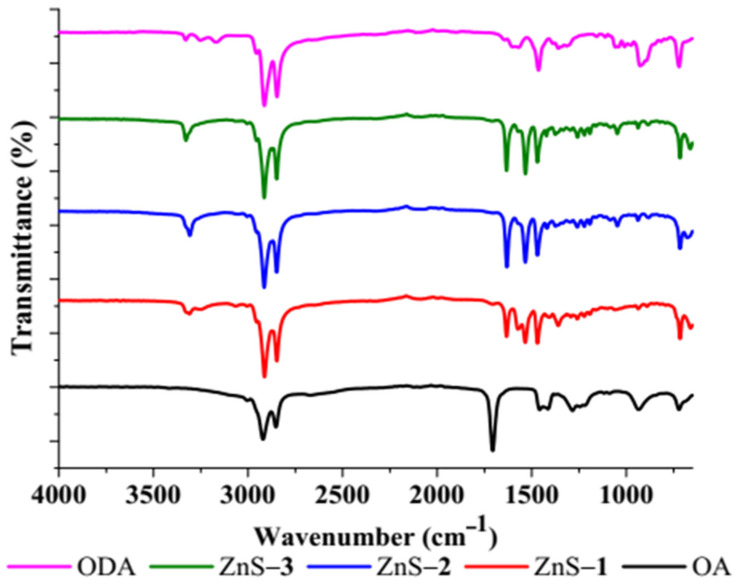
FTIR spectra of ZnS quantum dots.

FTIR spectra of the ZnS quantum dots (Figure 2) confirm the interactions of the octadecylamine-capping agent with ZnS. The C–C stretching vibrations of the capping agent appeared at 2853 cm^−1^ and 2907 cm^−1^, while the bands at 1367 cm^−1^ and 1466 cm^−1^ are attributed to C–H stretching vibrational modes [39]. ZnS stretching vibrations were observed at 1636 cm^−1^ and 1089 cm^−1^ [40,41,42], while the N–H absorption band was observed at 3330 cm^−1^, which confirms octadecylamine (ODA) as a capping agent on the surface of the ZnS quantum dots. The absence of C=O stretching frequency at 1700 cm^−1^ confirms that oleic acid (OA) is not present on the surface of the ZnS as a capping agent [43,44].

### 2.2. Optical Studies of ZnS Quantum Dots

**Figure 3:**
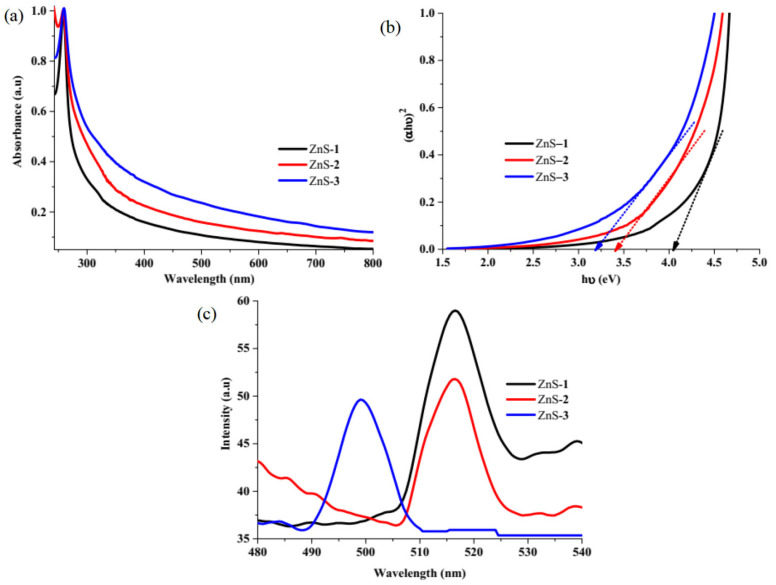
ZnS quantum dots absorption spectra (**a**), Tauc plots (**b**), and photoluminescence spectra (**c**).

Absorption spectra of the as-prepared ZnS quantum dots (Figure 3a) showed that absorption band edges of ZnS–**1** appeared at 303 nm, which were red-shifted in comparison to bulk ZnS (345 nm), while ZnS–**2** appeared at 358 nm, and ZnS–**3** appeared at 391 nm, which were blue-shifted; this could be ascribed to quantum confinement effect [45]. The optical band gaps estimated from Tauc plots (Figure 3b) were 4.09 eV for ZnS–**1**, 3.46 eV for ZnS–**2,** and 3.17 eV for ZnS–**3,** respectively. The energy band gap of ZnS–**2** and ZnS–**3** is lower than the band gap of bulk ZnS (3.63 eV), while that of ZnS–**1** is higher, which could be ascribed to quantum confinement effect. The photoluminescence spectra of the ZnS quantum dots measured at 350 nm excitation wavelength at room temperature (Figure 3c) showed narrow emission bands in the range of 480–540 nm. The emission spectra are dependent on an interfacial process, which occurs between the particle’s boundary and the surrounding medium [46,47]. The ZnS quantum dots emission maxima appeared at 517 nm for ZnS–**1** and ZnS–**2,** while that of ZnS–**3** appeared at 499 nm. These emission bands are associated with zinc vacancies and interstitial zinc atoms [12,40]. The increase in the emission spectra intensity resulted in the recombination of photogenerated electrons and holes [48,49]. ZnS–**1** and ZnS–**2,** with higher intensity, indicate a high recombination of photo-induced charge carriers, which could lead to low photocatalytic activity, whereas ZnS–**3,** with low intensity, is expected to have better separation efficiency, which could enhance its photocatalytic activity. The differences observed in the emission peaks of the ZnS quantum dots are similar to what has been reported previously [12,24].

### 2.3. Photodegradation of Brilliant Green and Rhodamine B Dyes by the ZnS Quantum Dots

#### 2.3.1. Effect of Catalytic Dosage on Photocatalytic Degradation

**Figure 4:**
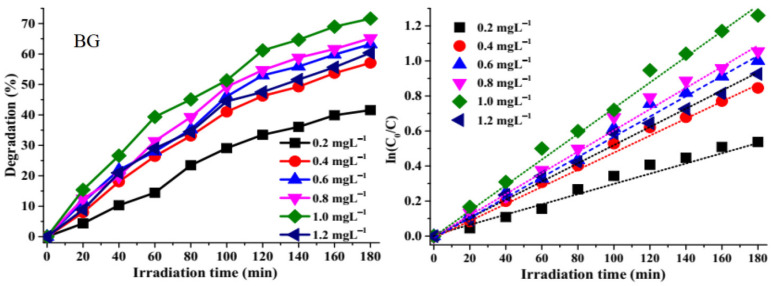
Effect of catalyst dosage on brilliant green over ZnS–**1** and corresponding In(*C_0_/C*) versus time plots.

In order to determine the optimum catalytic dosage of the ZnS photo catalyst for optimum photocatalytic degradation, the amount of ZnS quantum dots catalyst was varied from 0.2 mg L^−1^ to 1.2 mg L^−1^, while the concentration of brilliant green (BG) dye was kept constant at 5 ppm, as shown in Figure 4. The photocatalytic degradation efficiencies of BG and rate over ZnS–**1** increased from 41.57% (0.0032 min^−1^) for 0.2 mg L^−1^ to 71.61% (0.0071 min^−1^) for 1 mg L^−1^ after which it decreased slightly. This indicates that the increase in catalytic dosage results in higher degradation efficiency, which could be ascribed to the availability of active sites on the catalyst surface, which increases the generation of ·OH^−^ and ·O_2_^−^ radicals [50]. However, an increase in the catalyst dosage above 1 mg L^−1^ resulted in a decrease in photodegradation efficiency to 60.37% (0.0051 min^−1^) This could be ascribed to the scattering of light and the screening effect on the photocatalyst surface [51,52]. The results show that 1 mg L^−1^ is the optimal dosage for the photocatalytic degradation of the dyes. 

#### 2.3.2. Effect of Irradiation Time on Photocatalytic Degradation

**Figure 5:**
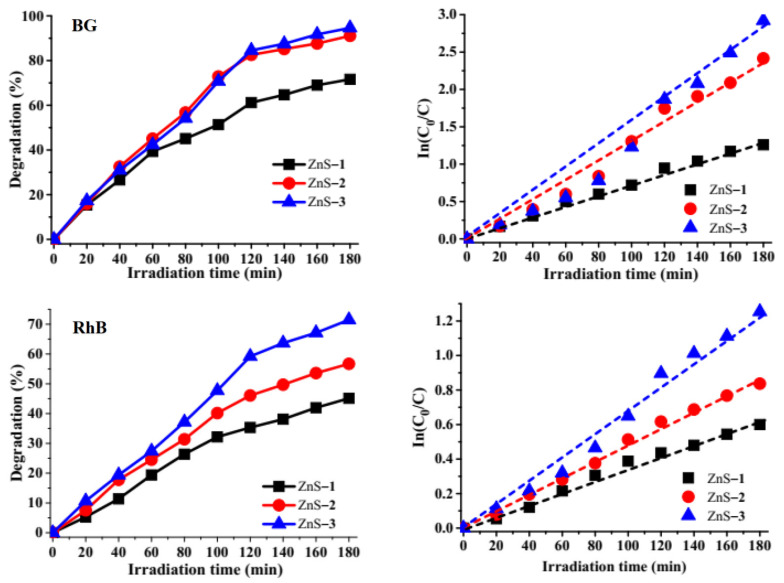
Degradation efficiency curves of brilliant green (BG) and rhodamine B (RhB) by ZnS quantum dots and their corresponding kinetics studies plot.

The photocatalytic degradation potential of the as-synthesized ZnS quantum dots were studied against brilliant green (BG) and rhodamine B (RhB) (singles dyes), and mixtures of both dyes (binary dye (BG-RhB)) in a solution (Figure S5). The photodegradation efficiencies of BG are 71.62% by ZnS–**1**, 91.06% by ZnS–**2,** and 94.61% by ZnS–**3** after 180 min, while the photo degradation efficiencies of RhB cationic dye are 45.12% by ZnS–**1**, 56.69% by ZnS–**2** and 71.46% by ZnS–**3**. The low degradation efficiencies of RhB by the as-prepared ZnS quantum dots could be attributed to its complex structure. The highest degradation efficiency was obtained from ZnS–**3** (Figure 5), which could be attributed to its small particle size and high rate of electron-hole pair separation efficiency, as observed in its photoluminescence spectra. While ZnS–**1** and ZnS–**2**’s lower degradation efficiency could be attributed to their high rate of electron-hole pair recombination. The overall high degradation efficiency observed by the ZnS quantum dots could be attributed to surface defects observed in the ZnS particles.

**Figure 6:**
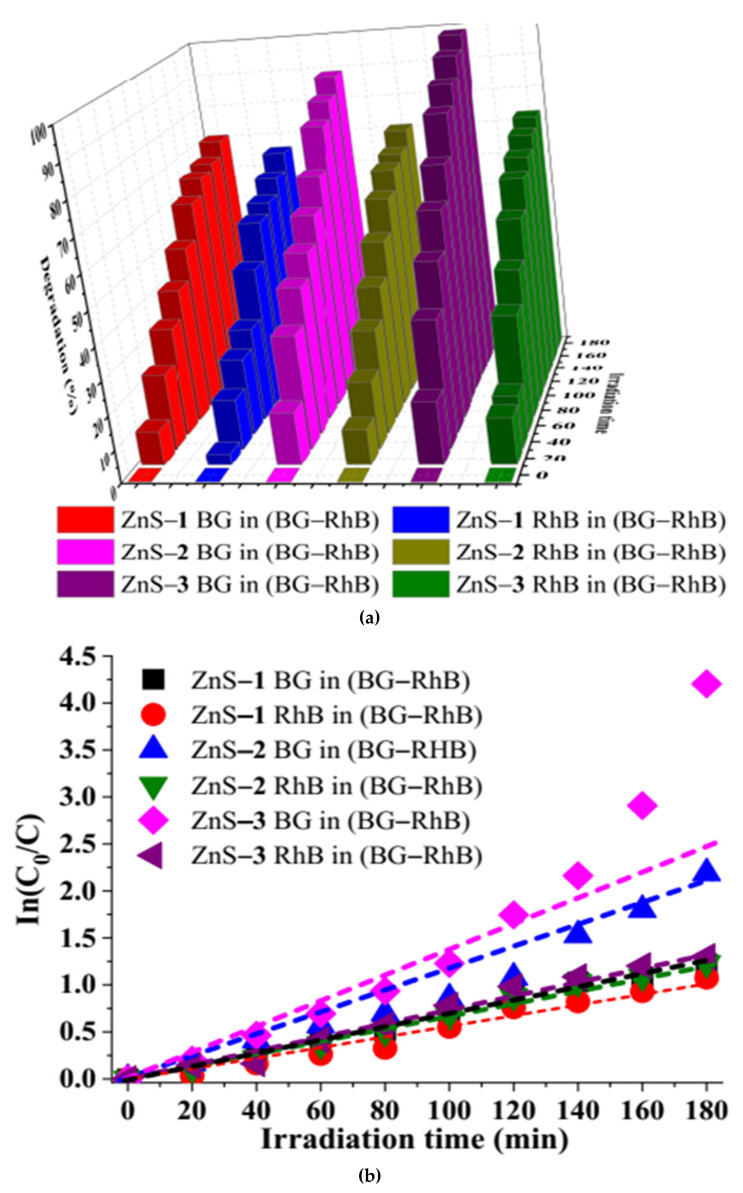
Degradation efficiency bar chart of binary dye by ZnS quantum dots (**a**), and corresponding kinetic studies plot (**b**).

The absorption spectra (Figure S6) of the binary-dye (BG–RhB) solution show that there is no overlap between the dyes. The absorption bands at 554 nm and 625 nm are ascribed to RhB and BG dyes, respectively. The photo degradation efficiency of BG in BG–RhB mixture is 70.60% by ZnS–**1**, 88.81% by ZnS–**2**, and 98.50% by ZnS–**3**, while the photo degradation efficiency of RhB in BG–RhB mixture was 65.81% by ZnS–**1**, 70.79% by ZnS–**2**, and 72.71% by ZnS–**3** (Figure 6). Overall, ZnS–**3** showed higher photocatalytic degradation of both the binary-dye mixture and single dyes, compared to ZnS–**1** and ZnS–**2**.

Analysis of the photodegradation of BG, RhB, and the binary dyes by the as-prepared ZnS quantum dots shows that the experiments followed pseudo-first-order kinetics due to the linear relationship between In(*C*_0_/*C*) versus irradiation time, as presented in Figure 5 and Figure 6. Equation (1) was used to determine the rate constant ‘*k*’ of the kinetics studies [53,54].

(1) $$\mathrm{In}\left(\frac{C_{0}}{C}\right)=kt$$

*C_0_* is the initial concentration at time ‘*0*’, while *C* is the concentration at a specific time and ‘*t*’ is the irradiation time. The extent of degradation and the corresponding rate constant and correlation coefficient are presented in Table 1.

#### 2.3.3. Effect of Scavengers on the Photocatalytic Degradation Efficiency of the Dyes

**Figure 7:**
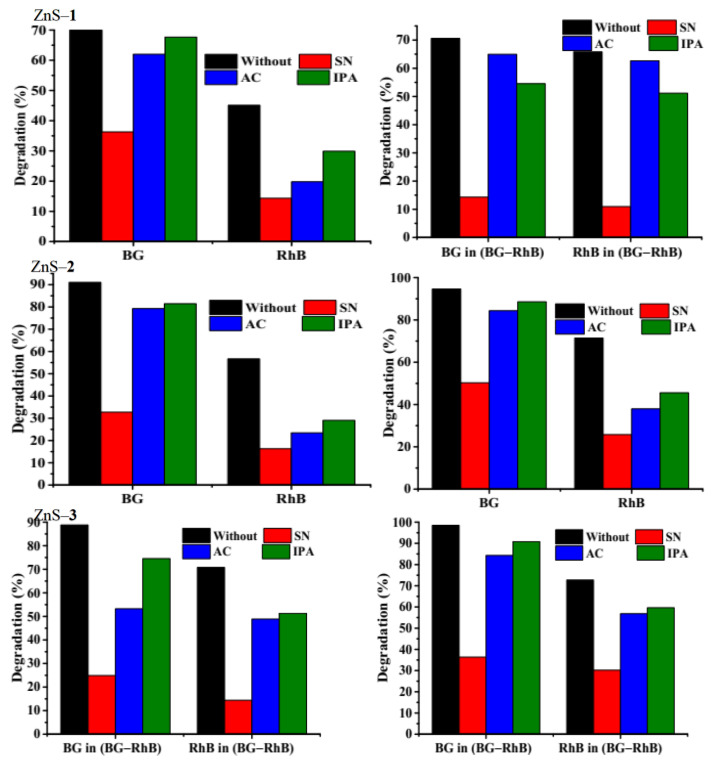
Effect of scavengers on *single and binary dyes over* ZnS quantum dots photocatalyst.

The photocatalytic degradation efficiency of any materials depends on the separation efficiency of electron-hole pairs (e^−^─h^+^) which lead to the generation of active species such as superoxide and hydroxyl radicals (·O_2_^−^ and ·OH^−^) through redox reactions. The photoluminescence spectra of the as-prepared ZnS quantum dots show defective state emission bands that promote lifetime separation of (e^−^─h^+^) pairs, which could enhance the photocatalytic degradation process. In order to establish which reactive species, influence the photodegradation process, isopropyl alcohol (IPA), silver nitrate (SN), and acrylamide (AC) were used as scavengers to quench ·OH^−^, e^−^, and ·O_2_^−^ [22,55,56]. A comparison between the photodegradation of brilliant green, rhodamine B, and the binary-dye solution after the addition of scavengers (Figure 7) indicates that brilliant green dye degradation efficiency by ZnS–**1** decreases from 71.62% (no scavenger) to 36.30% (SN), 61.99% (AC), and 67.65% (IPA) while its degradation by ZnS–**2** decreases from 91.06 to 32.72%, 79.26%, and 81.46%, and with ZnS–**3** it decreases from 94.61% to 50.28%, 84.37%, and 88.59%, respectively. This shows that the dominant active species are e^−^ and ·O_2_^−^, while ·OH^−^ acts as a secondary active species, which agrees with the trend observed with other catalysts [57,58]. However, the degradation efficiency of rhodamine B (RhB) by ZnS–**1** decreases from 45.12% (no scavenger) to 29.89% (IPA), 14.33% (SN) and 19.79% (AC) whereas with ZnS–**2** the degradation efficiency decreases from 56.69 (no scavenger) to 29.06% (IPA), 16.31% (SN) and 23.45 (AC). The effect of scavengers shows that with ZnS–**3,** the photocatalytic degradation efficiency decreases from 71.46% (no scavenger) to 45.56% (IPA), 25.78% (SN) and 37.97% (AC). The same trends were observed for the degradation of rhodamine B in the binary-dye mixture after the addition of scavengers. With the binary dyes, the addition of SN, AC, and IPA resulted in a reduction in degradation of RhB in (BG-RB) from 65.81% to 10.96%, 62.66%, and 51.17% by ZnS–**1**, while with ZnS–**2** the photocatalytic degradation efficiency decreased from 70.79% to 14.42%, 48.87%, and 51.29%, and with ZnS–**3** the photocatalytic degradation efficiency decreased from 72.71% to 30.20%, 56.82%, and 59.64%. However, for brilliant green dye in the mixed dyes, the degradation efficiency decreased from 70.60% to 14.32%, 64.94% and 54.56% by ZnS–**1**, while with ZnS–**2** it decreased from 88.80% to 24.91%, 53.23%, and 70.79%, and by ZnS–**3** the degradation efficiency decreased from 98.51% to 36.37%, 84.32%, and 90.81%, respectively. The results indicate that ·O_2_^−^ and e^−^ play significant roles in the degradation of rhodamine B compared to ·OH^−^, which agrees with previous reports using other photocatalysts [22,59].

#### 2.3.4. Photostability Studies of the ZnS Quantum Dots

**Figure 8:**
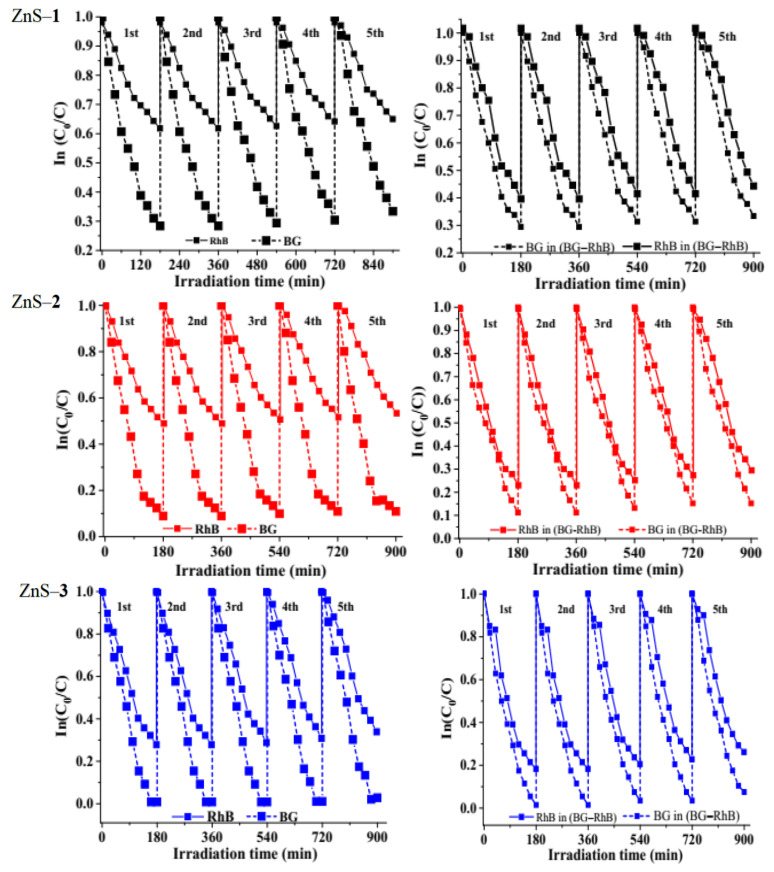
Reusability test of as-prepared ZnS catalysts on brilliant green (BG), rhodamine b (RhB), and binary dyes.

Photostability is an important attribute of photocatalysts. Hence, recyclability experiments were carried out to determine the stability of the as-synthesized ZnS. Figure 8 shows that the photodecomposition rates of brilliant green, rhodamine B, and binary dyes were significantly stable until the fifth cycles, which demonstrates excellent stability and reusability. After each run, the ZnS photocatalyst was washed by centrifugation and reused in the subsequent run. The reduction by the fifth cycle could be attributed to either accumulation of the by-products on the particles’ surface or the loss of particles during the filtration process, washing, and agglomeration of the catalyst as the number of cycles increases during the experiment [22,60].

#### 2.3.5. Effect of pH on Photocatalytic Degradation

It is important to evaluate the photocatalytic efficiency at different pH, as it is an important factor in changing the catalyst’s surface charge and the organic dye. It was observed that there was a sudden disappearance in color of brilliant green dye at pH 10, which could be due to the disruption of the alternate double- and single-conjugated bonds in brilliant green [61].

**Figure 9:**
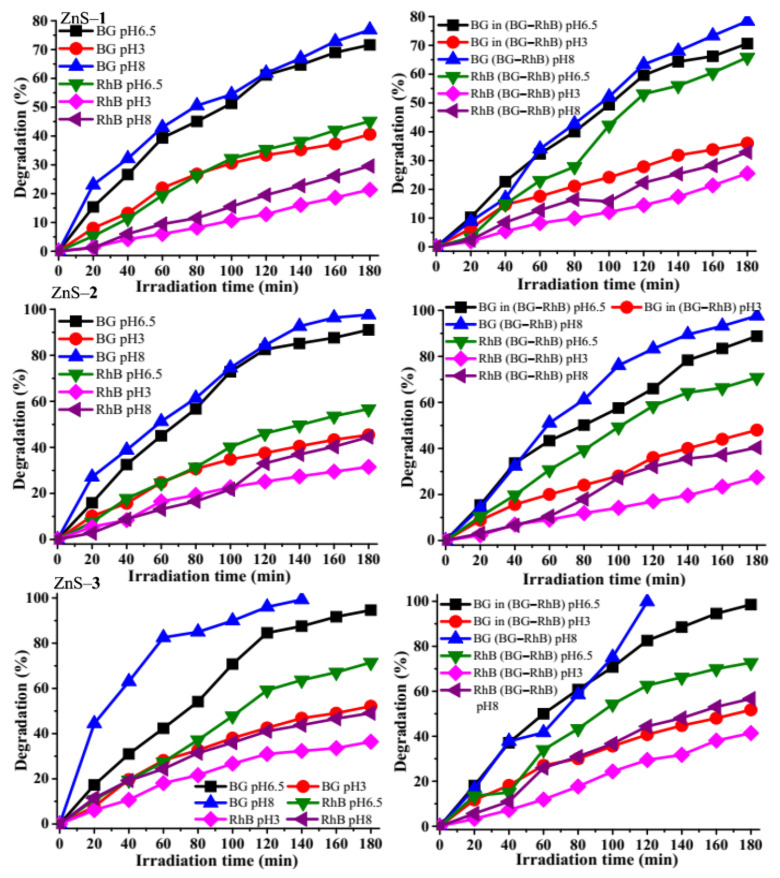
Effect of pH on brilliant green (BG), rhodamine B (RhB), and binary dyes over ZnS quantum dots.

It has been reported that at pH 10, the brilliant green dye becomes electrophilic, thereby becoming unstable and colorless [25], hence pH 8 was set as the maximum pH. Three different pH (3, 6.5, and 8) of the dye solution were used to evaluate the degradation efficiency at a constant catalytic dosage (5 mg) and dye concentration (5 ppm) for BG, RhB, and BG–RhB dye solutions (Figure 9). The degradation efficiency of the brilliant green solution by the ZnS quantum dots was at the lower pH 3 (40.57% for ZnS–**1**, 45.43% for ZnS–**2**, and 52.11% for ZnS–**3**), while at the higher pH 8, the degradation rate increased to 76.85% for ZnS–**1**, 97.64% for ZnS–**2**, and 99.29% for ZnS–**3**. The increase in the degradation efficiency could be attributed to the increase in availability of hydroxyl groups, making the degradation process favorable in alkaline media [57]. Furthermore, the degradation of rhodamine B using the same pH resulted in a different trend from brilliant green. At pH 3, the degradation efficiency of rhodamine B decreased to 21.35% for ZnS–**1**, 31.52% for ZnS–**2**, and 36.39% for ZnS–**3**. This could be attributed to the generation of Zn^2+^, which results in a reduced concentration of protons in the solution [62]. While at pH 8, the photocatalytic degradation efficiency increased to 29.60% for ZnS–**1**, 44.48% for ZnS–**2**, and 49.08% for ZnS–**3**. This could be due to the formation of rhodamine B zwitterion [63]. At pH higher than 8, rhodamine B has been reported to degrade poorly due to a high OH^−^ concentration which reacts with ·OH, reducing the availability of the active species [51]. The same trend was observed with the photocatalytic degradation of the binary dye. The mechanism of the brilliant green and rhodamine B is similar to other studies where tin sulfide and copper sulfide nanoparticles were used as photocatalysts [31,59].

## 3. Materials and Methods

### 3.1. Materials

Oleic acid (99%), octadecylamine (90%), methanol (≥99.9%), toluene (≥99.5%), brilliant green (~90.0%), rhodamine B (95%), silver nitrate (≥99.0%), acrylamide (98%), and isopropanol were purchased from Merck (Darmstadt, Germany). All reagents were used as obtained without further purification. Bis(morpholinyldithiocarbamato)Zn(II) complex was prepared using reported procedure [64]. 

### 3.2. Physical Characterization Techniques

Powder X-ray diffraction (XRD, Bruker D8 (Billerica, MA, USA) Cu-Kα irradiation λ = 1.5405 Å) was used for phase identification of the ZnS quantum dots. The surface morphology and elemental composition of the ZnS quantum dots were recorded with scanning electron microscope (ZEISS FEGSEM, Oberkochen, Germany) equipped with electron diffraction spectroscopy at 20 kV rating voltage. High-resolution transmission electron microscope (HRTEM, JEOL-2100 (Akishima, Tokyo, Japan)) was used to determine the particle size and shapes of the as-prepared ZnS nanoparticles. Agilent Cary 630 spectrometer (Santa Clara, CA, USA) was used to take FTIR spectra of the ZnS nanoparticles. PerkinElmer Lambda 25 UV-Vis spectrophotometer (Waltham, MA, USA) and PerkinElmer LS 45 fluorescence spectrometer (Waltham, MA, USA) were used to measure absorption spectra and photoluminescence, respectively.

### 3.3. Synthesis of ZnS Quantum Dots

Bis(morpholinyldithiocarbamato)Zn(II) complex (0.5 g) was dissolved in 15 mL of oleic acid; the suspension obtained was injected into octadecylamine (6 g) at 130 °C. A decrease in temperature to 117 °C was observed. The temperature was stabilized at 130 ℃ and maintained for 30 min under nitrogen flow while stirring. Afterward, the reaction mixture was cooled to 70 °C, and excess methanol was added to precipitate the nanoparticles. The nanoparticles were isolated by centrifugation at 5000 rpm for 30 min. The resulting solid was dried at room temperature and labeled ZnS–**1**. The complex was thermolyzed for 1 h to prepare ZnS–**2**, while thermolysis of the complex for 2 h resulted in zinc sulfide quantum dot labeled as ZnS–**3**.

### 3.4. Photocatalytic Studies of the ZnS Quantum Dots

The photocatalytic degradation efficiency of the ZnS quantum dots were evaluated through the photodegradation of brilliant green, rhodamine B and mixture of brilliant green-rhodamine B (binary dye) solution. Before irradiation, the ZnS quantum dots and the dye solution were stirred magnetically for 30 min under dark conditions to establish an adsorption–desorption equilibrium between dyes and photocatalyst surface. The study was carried out in vials under visible light irradiation. For the photo degradation experiment, 5 mL of brilliant green dye solution (5 ppm concentration) was treated with the different quantum dots (5 mg) in seven vials placed in circular metal support exposed to high pressure sodium lamp OSRAM VIALOX (80 W). A control experiment with ZnS quantum-dot-catalyst-free brilliant green solution was also carried out to monitor the photo degradation of the dyes. At regular time intervals (20 min), a sample vial was taken out and the concentration of the dye was measured by recording the UV absorbance in the range of 200–800 nm, using a UV-visible spectrophotometer.

The degradation efficiency was calculated from Equation (2) [65]:

(2) $$Efficiency\;(\%)\;=\;\frac{C_{0}\;-C}{C_{0}}\;\times 100$$

where *C*_0_ and *C* are the dye concentrations before and after photoirradiation, respectively.

ZnS quantum dots’ stability and reusability during the photocatalytic process were assessed with the same visible light irradiation and after each degradation cycle. The ZnS quantum dots’ photocatalyst was separated by filtration and washed many times with distilled water, and then photodegradation of the dyes was carried out under the same conditions.

### 3.5. Detection of Reactive Oxygen Species Test

The detection of reactive species is an essential theme in photocatalytic degradation process. Different scavengers such as isopropyl alcohol (IPA), acrylamide (AC), or silver nitrate (SN) were used as quenching agents of h^+^, e^−^ and OH^−^, respectively. Each scavenger was added into the brilliant green aqueous solution prior to the addition of ZnS quantum dots as photo catalysts. The exact process as mentioned earlier was repeated for aqueous rhodamine B and binary-dye solution [55].

## 4. Conclusions

Bis(morpholinyldithiocarbamato)Zn(II) complex was thermolyzed at 130 °C in octadecylamine at 30 min to prepared ZnS–**1**, at 1 h to prepared ZnS–**2**, and at 2 h to prepared ZnS–**3** quantum dots. The degradation of brilliant green, rhodamine B, and binary dye (mixture of brilliant green–rhodamine B) were used to evaluate the potential of the as-prepared zinc sulfide quantum dots as photocatalysts. Powder X-ray diffraction patterns confirmed the ZnS hexagonal wurtzite phase, and the diffraction peaks became sharper as the thermolysis time increased. HRTEM micrographs revealed spherically shaped particles with sizes of 1.3 nm for ZnS–**1** prepared at 30 min, 1.08–3.42 nm for ZnS–**2** prepared at 1 h, and 0.80–2.88 nm for ZnS–**3** prepared at 2 h. The energy band gap of the zinc sulfide quantum dots estimated from the Tauc plots were 4.09 eV for ZnS–**1**, 3.46 eV for ZnS–**2**, and 3.17 eV for ZnS–**3**. The energy band gaps were blue-shifted, which could be ascribed to quantum confinement effect as a result of the small particle size of the as-prepared ZnS quantum dots. The ZnS quantum dots were used as photocatalysts for the degradation of brilliant green (BG), rhodamine B (RhB), and a mixture of brilliant green and rhodamine B binary dyes (BG–RhB). Photocatalytic degradation efficiencies of 71.61% (ZnS–**1**), 91.06% (ZnS–2), and 94.61% (ZnS–**3**) were obtained for BG, while degradation efficiencies of 45.12% (ZnS–**1**), 56.69% (ZnS–**2**), and 71.46% (ZnS–**3**) were obtained for RhB. However, the degradation efficiency of BG in the binary-dye mixture (BG–RhB) by ZnS–**1** was 70.60%, 88.80% by ZnS–**2**, and 98.51% by ZnS–**3**, while the degradation efficiency of RhB in the ternary dye mixture (BG–RhB) was 65.81% by ZnS–**1**, 70.79% by ZnS–**2**, and 72.71% by ZnS–**3**. The studies show that degradation efficiency increases with an increase in catalytic dosage up to 1 mg mL^−1^ of the ZnS nano-photocatalyst, while alkaline medium is more favorable for photocatalytic degradation. The most dominant active species in the photodegradation of the dyes were electron holes, super oxides, and hydroxyl radicals. The reusability studies of the ZnS quantum dots indicate that they are stable and recyclable even after five catalytic cycles.

## Figures and Tables

**Table 1:** The photocatalytic degradation parameters of single and mixed dyes over ZnS quantum dots under visible light irradiation.

Dyes	ZnS Photocatalyst	Degradation Efficiency (%)	Rate Constant *k* (min^−1^)	R^2^
BG	ZnS–**1**	71.62	0.0071	0.994
	ZnS–**2**	91.06	0.0141	0.9854
	ZnS–**3**	94.61	0.0245	0.9197
RhB	ZnS–**1**	45.12	0.0034	0.9894
	ZnS–**2**	56.69	0.0048	0.9948
	ZnS–**3**	71.46	0.0073	0.9886
BG in (BG–RhB)	ZnS–**1**	70.79	0.0061	0.9921
	ZnS–**2**	88.81	0.0116	0.9659
	ZnS–**3**	98.51	0.0208	0.9045
RhB in (BG–RhB)	ZnS–**1**	65.81	0.0063	0.9818
	ZnS–**2**	70.60	0.0072	0.9928
	ZnS–**3**	72.71	0.0078	0.9855

## Supplementary Materials

The following are available online. Figure S1: Particle size distributions of the ZnS nanoparticles, Figure S2: Powder X-ray diffraction patterns of ZnS quantum dots prepared at 30 min (ZnS–**1**), 1 h (ZnS–**2**) and 2 h (ZnS–**3**), Figure S3: SEM images of the ZnS quantum dots, Figure S4: EDX spectra of the ZnS quantum dots, Figure S5: Absorption spectra of brilliant green (a–c) and rhodamine B (d–f) over ZnS quantum dots, Figure S6: Absorption spectra of BG–RhB degradation over ZnS quantum dots.
