# Supplementary material for: Photocatalytic Degradation of Single and Binary Mixture of Brilliant Green and Rhodamine B Dyes by Zinc Sulfide Quantum Dots
Source: Molecules. 2021 Dec 19;26(24):7686. doi: 10.3390/molecules26247686 (PMC8704525; doi:10.3390/molecules26247686)
Supplement: Supplementary file 1 [file molecules-26-07686-s001.zip › molecules-1485792-supplementary.pdf]

Photocatalytic degradation of single and binary mixture of brilliant green and rhodamine B dyes by zinc sulphide quantum dots

Peter A. Ajibade* and Abimbola E. Oluwalana

School of Chemistry and Physics, University of KwaZulu-Natal, Private Bag, Scottsville, Pietermaritzburg 3209, South Africa

Supplementary Information

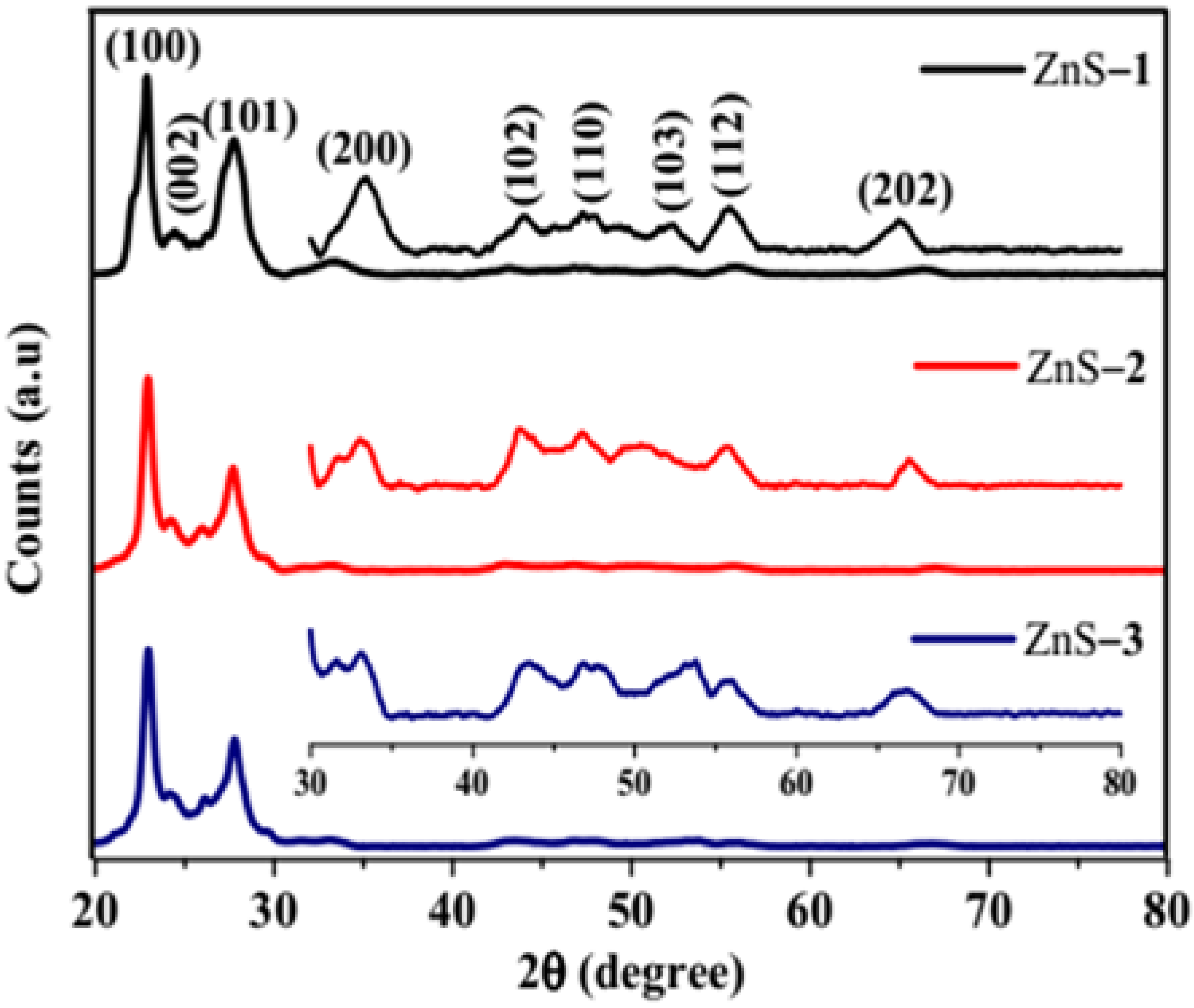

Figure S1: Powder X-ray diffraction patterns of ZnS quantum dots prepared at 30 min (ZnS–**1**), 1 h (ZnS–**2**) and 2 h (ZnS–**3**)

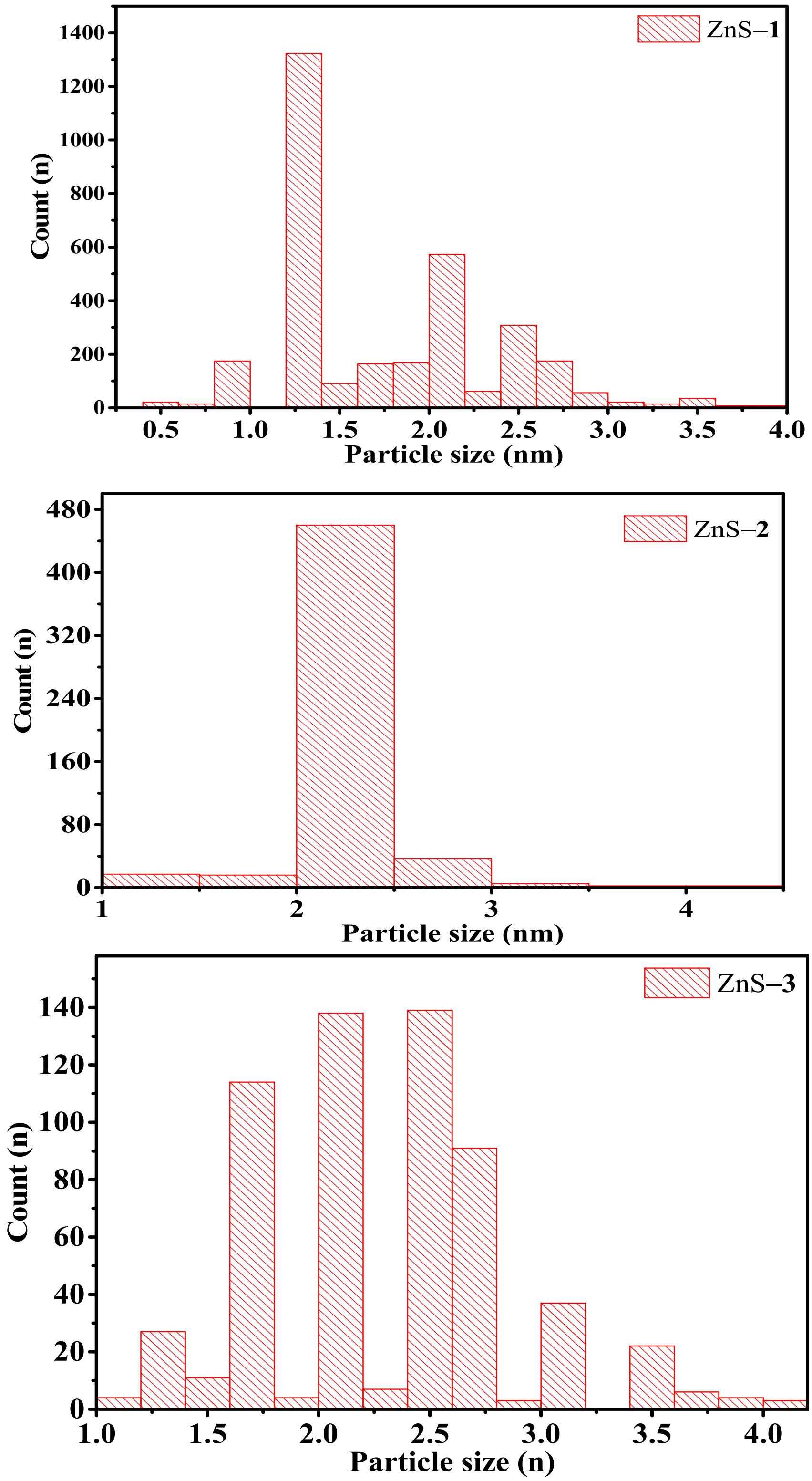

Figure S2: Particle size distributions of the ZnS nanoparticles

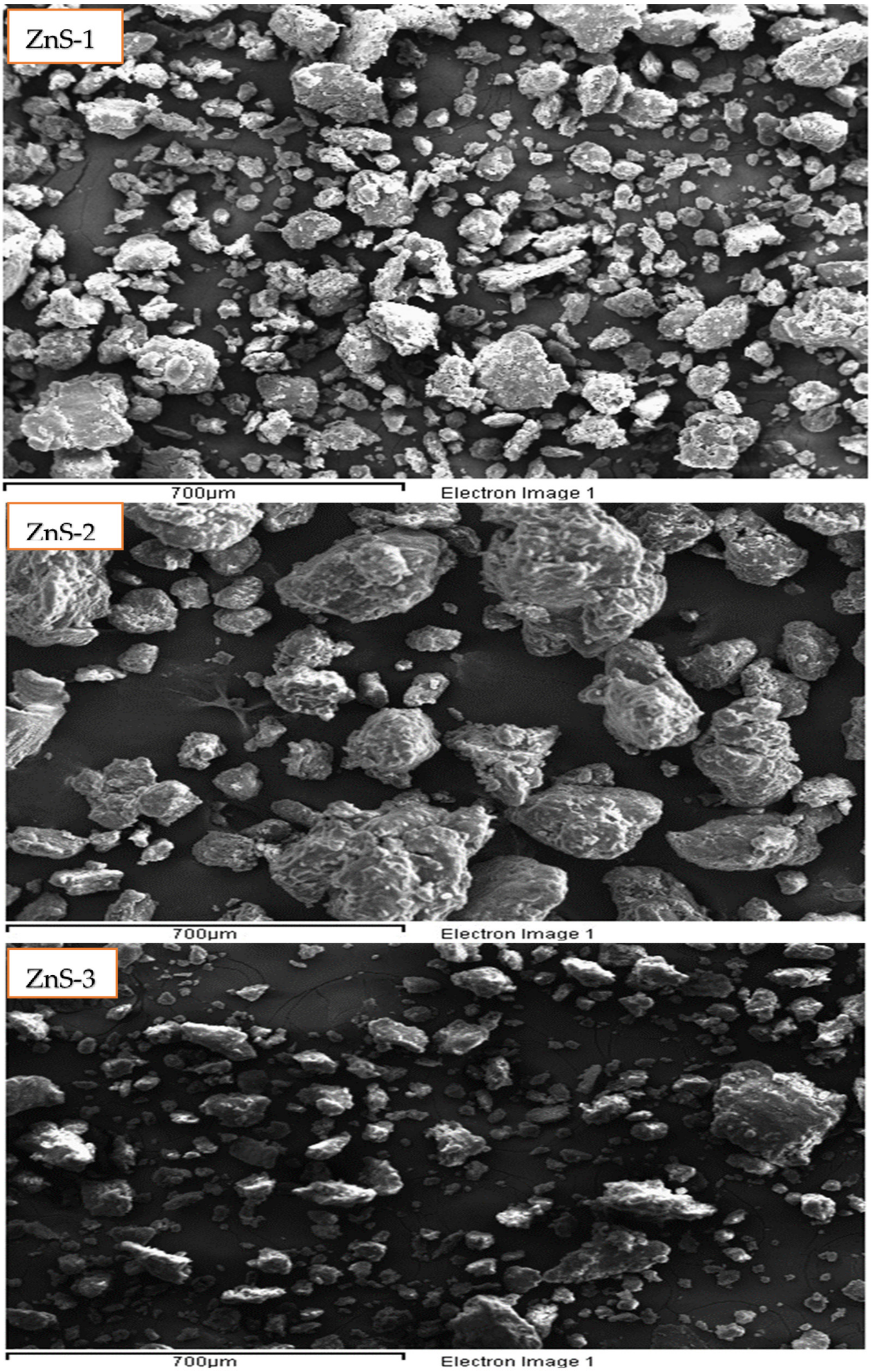

Figure S3: SEM images of the ZnS quantum dots

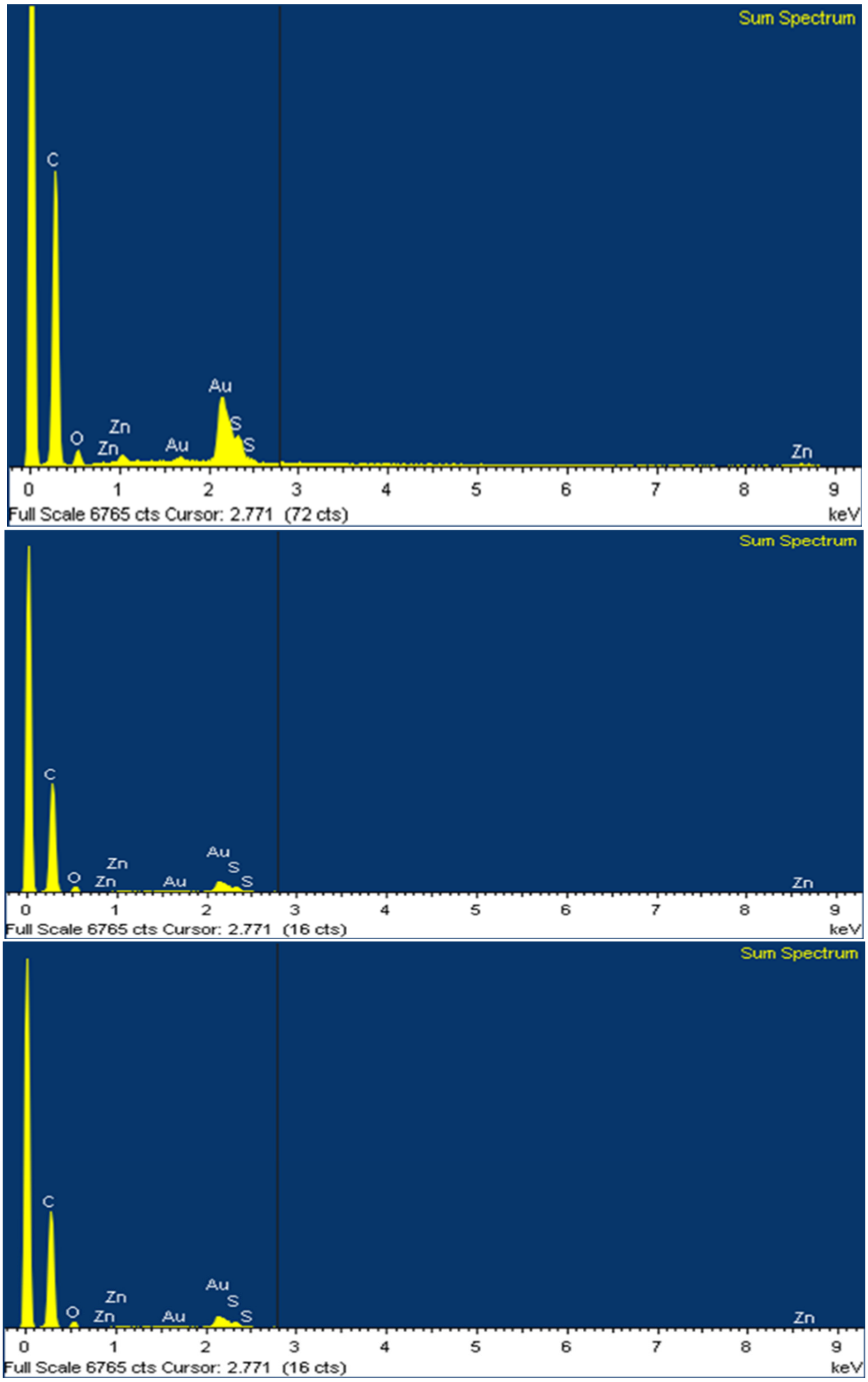

Figure S4: EDX spectra of the ZnS quantum dots

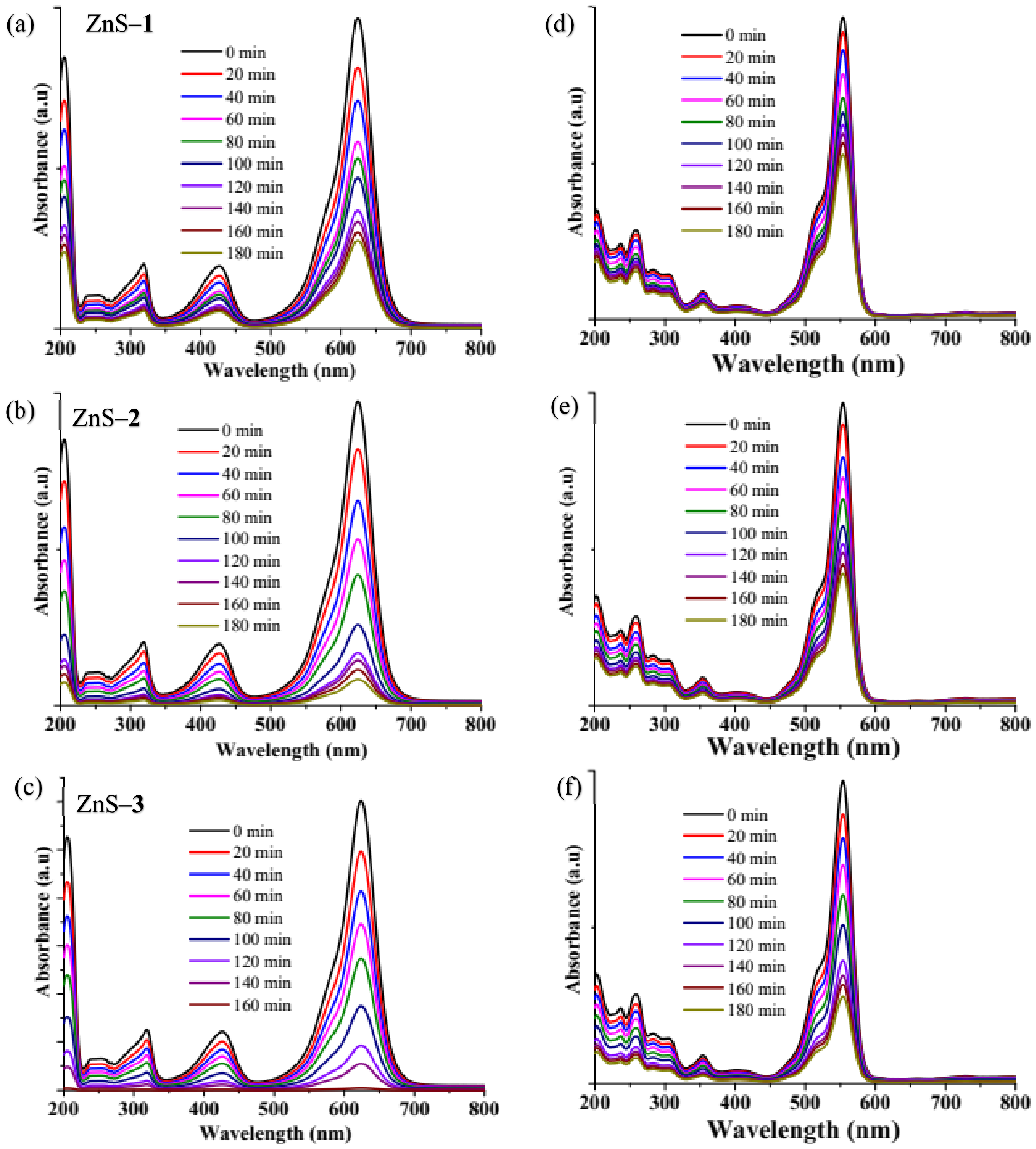

Figure S5: Absorption spectra of brilliant green (a-c) and rhodamine B (d-f) over ZnS quantum dots

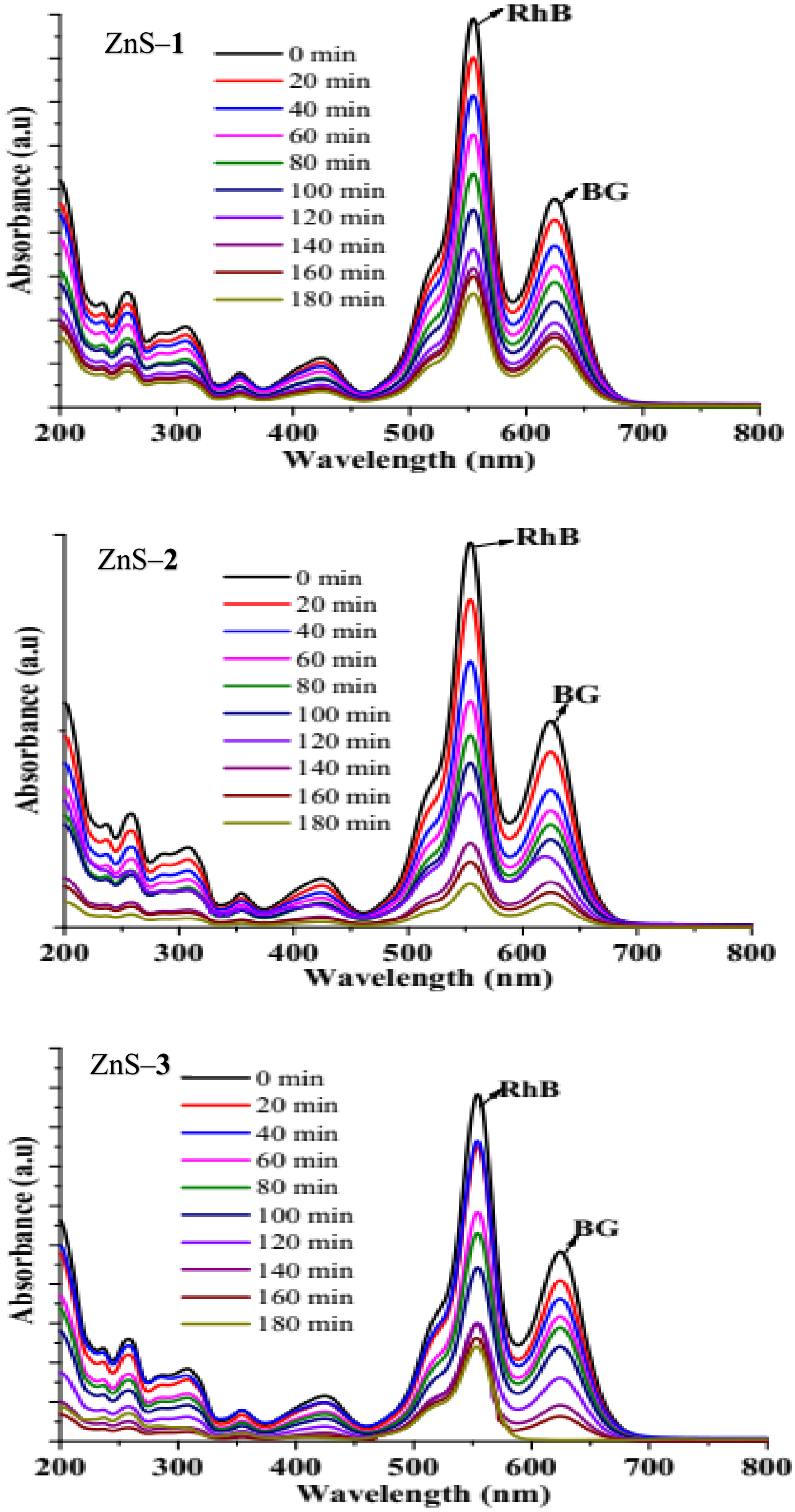

Figure S6: Absorption spectra of BG–RhB degradation over ZnS quantum dots
